# Exploring Physical Education Teachers’ Intention and Perceived Constraints in Offering Online Lessons Using the Theory of Planned Behavior: A Multi-Country Analysis

**DOI:** 10.3390/bs14040305

**Published:** 2024-04-09

**Authors:** Ferman Konukman, Bijen Filiz, Farhad Moghimehfar, Mona Adviento Maghanoy, Kim Graber, Kevin Andrew Richards, Christopher John Kinder, Yee Cheng Kueh, Ngien-Siong Chin, Garry Kuan, Gin Shi Jinyu

**Affiliations:** 1 Department of Physical Education, Qatar University, Doha 2713, Qatar; 2 Department of Coaching Education, Afyon Kocatepe University, Afyonkarahisar 03200, Turkey; bijenfiliz@aku.edu.tr; 3 Recreation and Tourism Management Department, Vancouver Island University, Vancouver, BC V9R 5S5, Canada; farhad.moghimehfar@viu.ca; 4 Department of Sports Science, College of Human Kinetics, University of the Philippinea, Diliman, Quezon City, Metro Manila 1101, Philippines; mamaghanoy@up.edu.ph; 5 Department of Kinesiology and Community Health, University of Illinois, 127 Freer Hall, 906 S. Goodwin Ave., Urbana, IL 61801, USAkarichar@illinois.edu (K.A.R.); ckinder3@illinois.edu (C.J.K.); 6 Biostatistics and Research Methodology Unit, School of Medical Sciences, Universiti Sains Malaysia, Kubang Kerian 16150, KTN, Malaysia; yckueh@usm.my; 7 Physical Education and Health Department, Institute of Teacher Education Batu Lintang Campus, Kuching 93200, SWK, Malaysia; ipgm-2785@moe-dl.edu.my; 8 Exercise and Sports Science, School of Health Sciences, Universiti Sains Malaysia, Kubang Kerian 16150, KTN, Malaysia; garry@usm.my; 9 Shanghai Tianyuan High School, Department of Physical Education and Sports, Shanghai University of Sports, Shanghai 200433, China; shijinyu2635@163.com

**Keywords:** physical education teacher, online education, theory of planned behavior, constraints, COVID-19

## Abstract

Guided by the theory of planned behavior, this study aimed to determine the influence of Physical Education (PE) teachers’ attitudes, their perceived behavioral control, and the influence of subjective norms on their intention and constraints (intrapersonal, interpersonal, and structural) to offer a high-quality class based on best practices to deliver PE lessons online during the COVID-19 pandemic. This cross-sectional, multi-country survey study recruited PE teachers from five countries (China, Malaysia, the Philippines, Turkey, and the United States). A total of 928 online questionnaires were used in the analysis. In terms of the overall intention to teach online, our findings showed that American and Filipino teachers had higher levels of intention to continue teaching online. In contrast, Turkish, Malaysian, and Chinese teachers showed a lower interest. Moreover, Malaysian teachers had more intrapersonal constraints while the teachers in the other four countries were not as restrained intrapersonally. The results highlight the significant influence of perceived behavioral control and attitudes on PE teachers’ intention to deliver online courses. Constraints to online teaching had a considerably large negative impact on attitudes, subjective norms, and perceived behavioral control. Based on the results, the proposed extension to the theory of planned behavior was an appropriate framework for understanding the behavioral intent of PE teachers.

## 1. Introduction

The COVID-19 pandemic is a global health pandemic affecting the daily lives of all citizens around the world. During this period, one of the most affected sectors in many countries was the K-12 school education system. Almost all countries had to make changes in their education systems, schools were closed, and the teaching process was carried out through online education platforms from March 2020 [1]. This situation caused teachers to alter their teaching style and pedagogical approach. Different countries implemented diverse practices regarding online education [2]. 

For the purpose of this study, the researchers evaluated the diversity of instructional online teaching practices for physical education teachers in the following countries: Turkey, the United States, the Philippines, Malaysia, and China. When reviewing the main policies in each country, we report on policies and actions reported by national governments and associations. In Turkey, the Ministry of National Education [3] offered online education to students via the Education Information Network (EBA) platform and EBA-TV. PE teachers implemented Physical Education (PE) lessons through Zoom (an online meeting application) and EBA platforms. In the United States, SHAPE America supported PE teachers by providing online virtual resources for health and physical education (#HPEatHome), including videos with practical and theoretical content, and most PE teachers conducted lessons in online or hybrid formats. In Malaysia, the Digital Educational Learning Initiative Malaysia (DELIMA) was created to provide resources, guidance, and information to students, teachers, and parents to adapt to online delivery formats. PE teachers used different applications, such as Google Meet and Microsoft Teams, to conduct online PE classes. In the Philippines, PE was shifted to an online platform. No face-to-face courses were held in compliance with the latest Omnibus Guidelines on the Imposed Community Quarantine in the Philippines. In China, video broadcasts were made on television, and online classes were held depending on the demand. Collaboration with parents was established by trying to strengthen the parent–student relationship by imposing responsibilities on parents. 

Overall, in most countries, the preparation of the content of PE lessons was left to the educators, who faced challenges linked to both their personal and professional responsibilities, worries about the welfare of their students that extended beyond educational matters, and difficulties with school administration and other organizational bodies regarding COVID-19 safety protocols [4]. There were concerns about teachers’ ability to make the pedagogical changes and decisions to still offer quality PE, while PE teachers were supposed to create self-made materials on physical education [5]. Although some studies’ results showed that students had a positive experience during online physical education courses [6], PE teachers’ intentions to develop online curricula and lessons is yet to be studied. Thus, understanding their perceived intentions and constraints would help researchers/practitioners to understand their ability to offer quality PE during this period. One way to explore intention is using the TPB. Guided by the theory of planned behavior (TPB) [7,8,9], the purpose of this study was to determine the influence of PE teachers’ attitudes, their perceived behavioral control, and the influence of subjective norms on their intention to deliver PE lessons online during the COVID-19 pandemic. Constraints to delivering PE lessons during the COVID-19 pandemic were also examined. The TPB was extended by including the hierarchical leisure constraints theory [10,11].

### 1.1. The Theory of Planned Behavior (TPB)

The TPB is one of the most successful frameworks for conceptualizing people’s participation in different activities [7,9,12]. The TPB has successfully conceptualized behavioral intent and behaviors in multiple areas, including several teaching-related studies as well as intentions to include technology in teaching [13]. The intention is the product of the individuals’ attitude toward a behavior, the influence of others (subjective norm) regarding that behavior, and the individuals’ perceptions of control over performing the behavior [14]. Attitude reflects how people positively or negatively value specific actions. Perceived behavioral control indicates the individuals’ perceptions of their self-efficacy and controllability to engage in activities. 

Studies using the TPB framework presented different findings regarding the theory’s predictive power. While some studies have emphasized the need for perceived behavioral control in the form of perception of institutional support, others have found that attitude was the best predictor of particular behaviors. For example, Kao et al.’s [15] survey of 358 elementary school teachers’ behavioral intentions regarding web-based professional development emphasized the importance of attitude. De Boer et al. [16] reported similar findings concerning the experience of regular primary school teachers implementing inclusive education. Holding a positive attitude was essential for such endeavors. 

**Figure 1:**
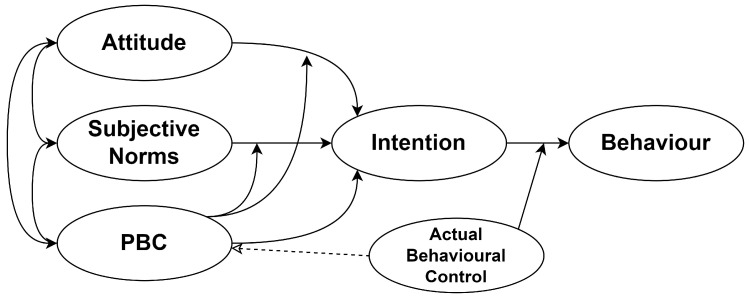
The theory of planned behavior (https://people.umass.edu/aizen/tpb.diag.html, accessed on 6 February 2024).

The TPB provides a clear structural model capturing the influence of the determinants of the behavior in different settings, including the acceptance of technology [17], such as in the case of this study [Figure 1]. Also, the TPB has proven effective in investigating different behaviors, namely teaching, e.g., [18,19,20,21,22]. However, the literature suggests the addition of new factors to extend the predictive power of the TPB [23]. For instance, in their review of the first decade of studies that employed the TPB as their framework, Connor and Armitage [24] supported the addition of new variables to the TPB. Since then, numerous studies have extended the theory by the adoption of other factors, such as motivation [25], beliefs [26], values [27], personal norms [28], constraints [29], negotiation of constraints [30], moral norms [31], worldview [32], self-identity [33], and perceived enjoyment [34]. The literature shows that these additions to the TPB have significantly improved its application in different fields of study. 

Technology-related behavior has been one of the research areas that has widely used the TPB as a framework to predict behaviors and behavior change [17], including the use of technology in teaching [13]. In response to the question “Can the TPB be expanded by adding more predictors of intention or behaviour?” Ajzen [17] (p. 317) indicated that “The TPB is, in principle, open to the inclusion of additional predictors. Just as the theory of reasoned action was expanded to produce the TPB by adding actual and perceived behavioural control, so too it is possible to include other predictor variables not already part of the theory”. A significant concept that can be added to the TPB is the restraining influence of perceived barriers. Although the TPB included perceived behavioral control, it does not consider the mitigating effect of constraints on intention. The recent literature has emphasized the importance of considering additional factors, such as personal limitations, environmental constraints, and unexpected barriers, to comprehensively understand why people act in the way they do [35]. The present study extended the TPB by adding these factors. 

### 1.2. Constraints to Online Teaching

Another theory that guided the framework of this study was the hierarchical leisure constraints theory [10,36]. This theory is utilized as an extension of the TPB in an effort to explain the influence of barriers on the decision-making process and participation of people in different activities [37]. This theory posits that, when deciding to participate in activities, particularly sports, leisure, and exercise participation, there are three types of constraints that influence people’s decisions: intrapersonal, interpersonal, and structural. These three categories of barriers restrain participation in a hierarchical order. First, intrapersonal constraints (i.e., lack of interest) limit an individual’s participation. Next, interpersonal constraints, such as family-led restrictions, restrain participation in the action. Finally, structural constraints, namely lack of infrastructure, prevent individuals from participating in the activity. Many studies have used the leisure constraints theory as an addition to the TPB. For example, Alexandris et al. [29] studied the behavioral intentions of fitness club members during COVID-19 restrictions. They extended the TPB by adding leisure constraints. Alexandris et al. [29] indicated the increasing importance of leisure constraints during the COVID-19 period. It is evident that, during the imposition of COVID-19 restrictions, social distancing regulations and norms (reflected as interpersonal constraints in the theory) became critical restraining factors influencing individuals’ well-being [38]. Also, limitations to the use of space (structural constraints), particularly for physical activities, dominantly limited people’s use of space during the pandemic. Therefore, participation in physical education activities is thought to have been significantly affected. These constraints also apply to the use of technology to teach such topics. Also, the period heavily influenced people’s personal approach to participating in physical exercise (intrapersonal constraints). These factors played a considerable role in people’s decision-making process regarding participating in physical activities. Alexandris et al. [29] found the TPB and leisure constraints theory as effective frameworks. Their results showed that attitude strongly influenced intention, followed by PBC and subjective norms. Other studies, such as that of Moghmiehfar et al. [30], extended the TPB by including leisure constraints. This study aims to extend the TPB by adding the leisure constraints theory’s factors (intrapersonal, interpersonal, and structural constraints) to explore the influence of these factors on PE teachers’ intention to teach online. 

Recognizing that each country has its own culture, challenges, and constraints, the use of this model in this multicultural context seems justified. An example of the application of this model in a multicultural context is Walker et al.’s [39] study of leisure and cultural constraints, in which they compared Canadian and Chinese university students. The findings showed that Chinese students had more interpersonal and interpersonal constraints, while Canadians perceived higher levels of structural constraints. In another study, the investigators examined the perceived constraints of 228 teachers in relation to leisure activities [32]; PE teachers were hindered more by personal factors, and non-physical education or non-sport teachers were more constrained due to social interactions. Kogler et al. [40] also used this theory in their research in a multicultural context during the COVID-19 pandemic to study people’s sport-related leisure behavior in Austria, Germany, and Italy. Other studies also investigated the barriers to teaching online during the COVID-19 pandemic [41]. 

In summary, as a result of COVID-19 restrictions, the necessity for innovative physical education delivery is critical. With the area of responsibility falling on physical educators, there is a need to examine the decision-making process of these teachers concerning generating strategies to deliver physical education remotely and effectively. This study considers the intentions of PE teachers to teach online vis-a-vis the cultural context and the accompanying constraints that come with each participating country.

### 1.3. Purpose Statement

**Figure 2:**
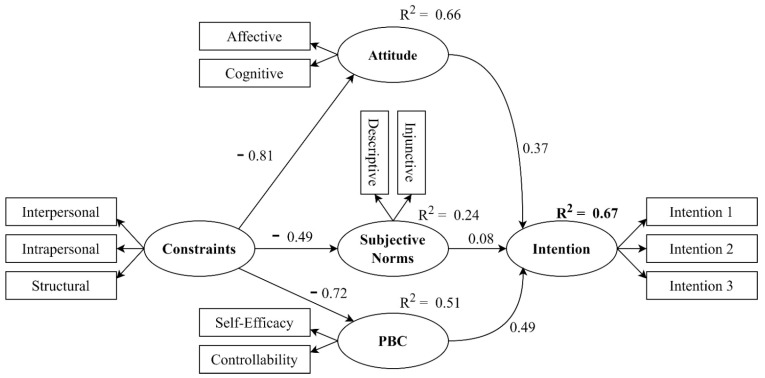
Structural model.

This study proposed a structural model based on the TPB [7,8] to examine the influence of PE teachers’ attitudes, perceived behavioral control, and the impact of subjective norms on their intention to offer a high-quality class based on best practices to deliver online physical education lessons during the COVID-19 pandemic. We included intrapersonal, interpersonal, and structural constraints to investigate PE teachers’ barriers and enhance the structural model’s predictability. We proposed that subjective norms, attitudes, and perceived behavioral control positively and directly influenced the intention of PE teachers to teach online courses. We also hypothesized that constraints on teaching online directly and negatively affected subjective norms, attitudes, and perceived behavioral control and indirectly and negatively impacted intentions (Figure 2).

## 2. Materials and Methods

### 2.1. Research Design and Participants 

A collaborative research group was established to conduct this study, including five research teams from China, Malaysia, the Philippines, Turkey, and the United States. This cross-sectional, multi-country survey study recruited PE teachers from five different countries. 

### 2.2. Sample

The sample included PE teachers from China (*n* = 153), Malaysia (*n* = 141), the Philippines (*n* = 67), Turkey (*n* = 215), and the United States (*n* = 352). All participants were over the age of 23 years, with the majority being under 40 years old (62%). More than 56% of the PE teachers identified as female, 43% as male, and 0.5% as of non-binary gender. Of the participants, 77% worked at state (public) schools and 7.4% at private schools. More than 40% were middle school teachers, followed by 25% high school and 22% primary school. Nearly 52% had less than ten years of experience teaching physical education (Table 1). 

### 2.3. Process

The researchers sent an invitation to participate in the online survey to PE teachers via social media and e-mail accounts (e.g., WhatsApp, Facebook, and WeChat). The first page of the survey included the study eligibility criteria: PE teachers working in a private or public school and those working in a secondary or high school, and PE teachers with experience teaching physical education online. The survey took about 20 min to complete. Data collection was carried out in each country between November 2020 and March 2021. Due to the pandemic, schools in the research countries delivered lessons remotely between these dates.

### 2.4. Instruments

A 15-item scale focusing on the teachers’ intention to teach physical education courses online was developed to investigate the associations proposed in the TPB. The items were based on Ajzen’s [7] and Francis et al.’s [42] guidelines and Moghimehfar et al.’s [30] study. Teachers’ intention was measured using three items focused on teachers’ behavioral intention and willingness to continue online teaching. Four items were designed to capture injunctive and descriptive subjective norms. The PE teachers’ perception of self-efficacy and controllability (i.e., perceived behavioral control) was measured with four items. Finally, four items were developed to measure the participants’ cognitive and affective attitudes toward teaching online (see Table 2).

Constraint items were developed based on previous leisure and social psychology studies, i.e., [29,43,44]. These items were modified to fit the context of this research (constraints to teaching PE online during the COVID-19 pandemic). A total of 12 items were used to investigate the perceived constraints to teaching physical education courses online. These were categorized as intrapersonal (three items), interpersonal (three items), and structural constraints (six items) based on the hierarchical leisure constraints theory [10]. A five-point Likert scale (from 1 = strongly disagree to 5 = strongly agree) was used to measure these items.

The initial survey was designed in the English language. The three countries that used non-English versions of the survey (i.e., Chinese, Malay, and Turkish) translated the items; then, Brislin’s study [45] was used to back translate the surveys by independent bilingual professionals to ensure accuracy. All the items in this research were previously tested and validated in numerous studies in different contexts, including education and sports sciences. 

We conducted a confirmatory factorial analysis (CFA) to validate the data across groups for the TPB variables as well as the constraints. The variables were used in different contexts. The items in our model had factor loadings greater than 0.50 (two items showed a factor loading between 0.50 and 0.60, and the rest of the items’ loadings were greater than 0.60), confirming the convergent validity of the structural model. The goodness-of-fit indices for the overall CFA analysis indicated an adequate model–data fit, with a chi-squared ratio (χ^2^/df = 2.67) of 273 and IFI = 0.95, NFI = 0.93, GFI = 0.93, and CFI = 0.95 close to 1 as well as RMR and RMSEA values close to zero (0.047 and 0.084, respectively; PCLOSE = 0.002). Cronbach’s alpha coefficient was used to examine the internal consistency of the items. All the constructs showed an acceptable internal consistency, excluding perceived behavioral control (0.66) and intrapersonal constraints (0.65). The low values obtained for these two constructs are thought to be due to cultural differences (Table 2).

### 2.5. Data Analysis

Data analysis began with the standard procedures for data cleaning and screening [46]. No data were extracted from the dataset because of the lack of extreme values that would affect the data analysis. Frequency and percentage analyses were used for the demographic information. In addition, frequency and percentage analyses were conducted to determine the participants’ employment status before and during the pandemic, their experience of teaching physical education and sports online before the pandemic, and their teaching preferences. A one-way ANOVA was conducted to identify differences in PETs’ overall intentions, attitudes, subjective norms perceived behavioral control, and constraints to teaching online. Tukey’s HSD post hoc test was used to explore these differences. Structural equation modeling was conducted to determine the relationship between the PETs’ intentions toward online teaching and the factors affecting it. The overall model fit, preliminary fit criteria, and fit of the internal structure of models were tested using [47] the incremental fit index (IFI), normed fit index (NFI), comparative fit index (CFI), goodness-of-fit index (GFI), root-mean-square error of approximation (RMSEA), and root-mean-square residual index (RMR). IBM Amos 24.0 and SPSS 26.0 were used to analyze the data. 

## 3. Results

### 3.1. Teaching Experiences before and during the COVID-19 Pandemic

The respondents were asked to report their employment status before and during the COVID-19 pandemic. A significant number (91%) were employed full-time before the pandemic, and about 75% did not experience any changes in their employment status during the pandemic. Nearly 70% of the respondents had no experience teaching physical education topics online before the pandemic. However, 82% indicated that they started teaching online during the period of global COVID-19 restrictions. When the individuals were asked about their teaching preference (online vs. face-to-face), nearly 75% indicated that they preferred to teach face-to-face or hybrid (a combination of face-to-face and online teaching) (Table 3). 

### 3.2. One-Way ANOVA

A one-way ANOVA was conducted to investigate the between-group differences in the sample. The Fisher’s test results show significant differences among the five countries. Tukey’s HSD post hoc test was used to explore these differences. Regarding the teachers’ overall intention to teach online, the results show that American and Filipino teachers had higher levels of intention to continue teaching online (mean > 3.70), whereas Turkish, Malaysian, and Chinese teachers showed a lower interest (3.05 < mean < 3.25). Turkish teachers showed a lower overall positive attitude toward teaching online (mean = 2.66), whereas Filipino (mean = 3.51) and Chinese (mean = 3.32) teachers showed a more positive attitude. Subjective norms had a higher impact on American (mean = 3.36) and Filipino (mean = 3.37) teachers compared to Turkish (mean = 3.02), Chinese (mean = 2.97), and Malaysian (mean = 3.14) teachers. Turkish teachers perceived a higher level of control over teaching online (mean = 3.31) compared to teachers from the other four countries (2.10 < mean < 3.01). 

Malaysian teachers perceived a higher number of intrapersonal constraints (mean = 3.05), whereas teachers from the other four countries were not as restrained intrapersonally (2.20 < mean < 2.40). Turkish, American, and Chinese teachers had fewer perceived interpersonal constraints (2.73 < mean < 2.82), whereas Filipino teachers (mean = 2.33) had the lowest and Malaysians (mean = 3.18) had the highest levels of perceived interpersonal constraints. Finally, Turkish (mean = 2.55) and Chinese (mean = 2.66) teachers faced fewer structural constraints than teachers from the other three countries (Table 4). 

### 3.3. Regression Associations

Overall, the model explained 67% of the variation in intention (R^2^ = 0.67). The RMSEA was 0.08 (values close to 0.05 are suggested as a good fit), and the RMR was 0.043 (an RMR value smaller than 0.05 reflects a good fit). The IFI, NFI, GFI, and CFI for this model were all close to 0.95, which is considered a good fit [48]. The model showed a strong fit with the data (Table 5). 

### 3.4. Structural Model

The structural modeling results (Table 6; Figure 2) showed that the attitude toward teaching physical education online, the influence of others (subjective norms), and teachers perceived behavioral control directly and positively influenced intentions. Of these significant associations, perceived behavioral control showed the strongest influence on intention (β = 0.49, *p*-value < 0.001), followed by attitude (β = 0.37, *p*-value < 0.001). Although significant, subjective norms did not show a considerable association with intention (β = 0.08, *p*-value < 0.05). 

Constraints (intrapersonal, interpersonal, and structural) showed a significantly large negative impact on attitude (β = −0.81, *p*-value < 0.001), subjective norms (β = −0.49, *p*-value < 0.001), and perceived behavioral control (β = −0.72, *p*-value < 0.001). The total indirect effect of constraints on intention was 0.69. 

## 4. Discussion

This study investigated the influence of PE teachers’ attitudes, perceived behavioral control, and the influence of subjective norms on PE teachers’ intention to deliver a high-quality PE lesson based on best practices online during the COVID-19 pandemic. A structural model based on the theory of planned behavior was used [7,8]. The study also included intrapersonal, interpersonal, and structural constraints in the framework to enhance the model’s predictability. 

We asked the respondents to report their employment status before and during the pandemic. Most PE teachers were employed full-time before the pandemic and did not have any experience of online education. Most stated that they started online education during the pandemic. In addition, most of the PE teachers said that they prefer face-to-face or hybrid instruction. Overall, participants did not prefer the online teaching of PE lessons. There may be various reasons for this, such as the difficulty of applying physical activity in online education, the inability to access appropriate course resources, and students’ indifference to online education. In their study, Korcz et al. [49] determined that, while teachers in some countries approached online education positively (Poland, Croatia, and Bulgaria), teachers in other countries approached it negatively (Turkey, North Macedonia, and Kosovo).

The present study found significant differences among the five countries. In terms of the overall intention to teach online, our findings showed that American and Filipino teachers had higher levels of intention to continue teaching online. In contrast, Turkish, Malaysian, and Chinese teachers showed a lower interest. Turkish teachers showed a lower overall positive attitude toward teaching online, whereas Filipino and Chinese teachers showed a more positive attitude. Subjective norms had a higher impact on American and Filipino teachers compared to Turkish, Chinese, and Malaysian teachers. Turkish teachers perceived a higher level of control when teaching online compared to teachers from the other four countries. In one study, PE teachers stated that organizing online learning was presented to them as a new work experience [50]. In addition, PE teachers stated that the motor skills of the students deteriorated during online education [51,52]. Also, Korcz et al. [49] stated that PE teachers pointed out the significant negative consequences of online PE, such as limited contact with pupils and a lack of control over the quality of the teaching process for schools.

In the present study, Malaysian teachers perceived more intrapersonal constraints, while the teachers from the other four countries were not as restrained intrapersonally. However, Turkish, American, and Chinese teachers had fewer perceived interpersonal constraints, whereas Filipino teachers had the lowest and Malaysians had the highest levels of perceived interpersonal constraints. Finally, Turkish and Chinese teachers faced fewer structural constraints compared to the teachers from the other three countries. For online education, factors such as a suitable environment, technical equipment, students’ willingness, teachers’ readiness for online education, and providing the necessary school and government support are effective [50]. Failure to provide these factors at a sufficient level may cause a sense of restriction. The fact that there are different approaches to online education in the countries that were the research subject may have caused teachers to feel different levels of restraint.

This study included PE teachers who worked at different teaching levels and type of schools in five countries (i.e., China, Malaysia, the Philippines, Turkey, and the US). The present study was limited to teaching physical education online (guided by the TPB and leisure constraints theory). We found the extended TPB suitable for understanding the behavioral intent of PE teachers during online education. An avenue for future research is to compare the results from PE teachers with those of other fields to better understand how online teaching influenced education systems.

The structural equation modeling results indicate that attitude toward teaching PE online, the influence of others (subjective norms), and teachers’ perceived behavioral control directly and positively influence the teachers’ intention to teach PE online. Of these significant associations, perceived behavioral control strongly influences intention, followed by attitude. Although significant, subjective norms do not show a considerable association with intention. Moreover, constraints (i.e., intrapersonal, interpersonal, and structural) show a significant negative impact on subjective norms, attitude, and perceived behavioral control. Dunn et al. [53] used the application of the TPB in examining the factors related to the teachers’ intentions to engage in ongoing professional training. Data were collected from 152 teachers learning Mathematics Common Core State Standards (CCSS). Their findings showed that intention was predicted significantly by perceived behavioral control, subjective norm, and attitude toward the behavior. It was perceived behavioral control that was the strongest predictor of intention. This represents the presence or absence of support from the institution to which the teachers were affiliated. 

Teo [54] conducted a study to examine the factors that explain the teachers’ intention to use technology. The relationship among perceived usefulness, ease of use, subjective norm, facilitating conditions, attitude toward use, and behavioral intention to use technology were investigated. The results revealed a good model fit. Employing the TPB as their framework, Smarkola [55] conducted a qualitative research study to investigate the intention to use computer devices in teaching. The results affirmed that intentions lead to action. The participants indicated that their main motivation for using computers was to help students to have real-world experiences. The teachers used both equipment and support to integrate these practices into training. Such findings reiterate the need for support and demonstrate that perceived behavioral control influences their behavior and decision-making process. Our study’s findings support their results.

## 5. Implications

Although some studies explored the experiences of PE teachers during the COVID-19 pandemic in a cross-cultural context [56] and the role of technology in the success of PE teachers [57], the challenges and barriers [41] and the impact of such practices still needs to be empirically tested. The results from this study indicate that attitude toward teaching physical education online, the influence of others (subjective norms), and teachers’ perceived behavioral control directly and positively influence intentions. Of these significant associations, perceived behavioral control shows the most substantial influence on intention, followed by attitude. Since perceived behavioral control is the most important factor directly influencing intention, it is important to facilitate the process of enabling PE teachers to believe they are in control of their behavior. Institutions can support PE teachers by helping them to set personal goals aligned with the institutions’ goals. Developing educational programs for PE teachers that introduce them to technologies and logistics is critical due to the practical nature of physical education. This will improve PE teachers’ self-efficacy and controllability (perceived behavioral control), which was the most important factor in our research (perceived behavioral control in the theory of planned behavior refers to two factors: controllability over decision-making processes and self-efficacy). Providing counseling and technology education and support that improve PE teachers’ self-efficacy and autonomy when teaching PE online are effective plans that institutions can implement to improve the quality of PE during restrictions. 

In determining outcome goals (i.e., performance and process), PE teachers may have a more precise roadmap of how to accomplish the challenging task of teaching physical education in situations where face-to-face interactions are not possible. By having clear goals and eventually accomplishing them, teachers have more evidence of their competence, which can facilitate the perception of being in control of their behavior, leading to positive outcomes.

Our results show that constraints on teaching physical education online directly and negatively influenced attitude, subjective norms, and perceived behavioral control and indirectly and negatively influenced intentions. Knowing that constraints largely influenced subjective norms, attitude, and perceived behavioral control negatively, it would be ideal for institutions to implement strategies to remove such constraints. Since each country had differences in the type of constraint that had the highest impact, it would be helpful to look at and understand the cultural influences of constraints and see how they can be removed or facilitated. Institutions may benefit from knowing what their structural constraints are. Mitigating these constraints facilitates the influence of attitude, perceived behavioral control, and intention. The significantly large impact of constraints on PE teachers’ attitudes and perceived behavioral control indicates that removing intrapersonal, interpersonal, and structural barriers can significantly improve attitudes and perceived behavioral control. Strategies include improving technology-use-related skills, providing online tools and teaching techniques, motivational workshops, and mental support (intrapersonal constraints). Developing support groups for PE teachers where they can learn from other PE teachers’ experiences and serve as motivation may remove some of the interpersonal barriers. All of these are possible when the right technology and technical support are provided (structural barriers).

In summary, technology will be a very important factor for all teachers in delivering instruction in the 21st century. PE teachers will be part of this trend as well. Therefore, PE teachers should improve themselves in this field. Public and private schools have limited time and resources for physical education. Considering the recent obesity epidemic, each PE teacher should be a personal coach and mentor for students. PE teachers can effectively deliver and manage this duty using online instruction and instructional technology as personal coaches and mentors. Thus, PE can extend beyond the gym and school borders in an innovative way. 

## 6. Conclusions

The present study’s findings supported our thinking that constraints to teaching online PE directly and negatively influence subjective norms, attitudes, and perceived behavioral control and indirectly and negatively influence intentions in five different countries around the world. Based on the results, the TPB is an appropriate framework for understanding the behavioral intent of PE teachers. 

## Figures and Tables

**Table 1:** Demographic characteristics of the respondents.

Variable	Frequency	Percentage
**Gender**		
Women	521	56.1
Men	402	43.3
Non-binary	5	0.5
**Age**		
Under 30 years old	261	28.1
31–35 years old	153	16.5
36–40 years old	167	18
41–45 years old	110	11.9
46–50 years old	114	12.3
51–60 years old	116	12.5
60+ years old	7	0.8
**Nationality**		
China	153	16.5
Malaysia	141	15.2
Philippines	67	7.2
Turkey	215	23.2
United States	352	37.9
**Type of school**		
State	719	77.5
Private	69	7.4
Foundation	2	0.2
Other	138	14.9
**Teaching level**		
Nursery school	3	0.3
Primary school	206	22.2
Middle school	385	41.5
High school	237	25.5
Tertiary	67	7.2
Other	30	3.2
**Years of professional service**		
1–5 years	284	30.6
6–10 years	198	21.3
11–15 years	137	14.8
16–20 years	105	11.3
21 and above	2.4	22

**Table 2:** Time allocation for teaching physical education courses.

Variables	Frequency					Mean	*SD*
**Section 1: Theory of Planned Behavior** **Intention (α = 0.83)**	**1**	**2**	**3**	**4**	**5**		
I expect myself to continue teaching physical education online during the COVID-19 period	64	109	167	335	253	3.65	1.19
I want to continue teaching physical education online during the COVID-19 period	135	155	216	261	161	3.17	1.30
I intend to continue teaching physical education online during the COVID-19 period	56	124	222	363	163	3.49	1.11
**Attitude (α = 0.70)**							
Unfulfilling–Fulfilling	119	144	346	215	104	3.04	1.16
Unpleasant–Pleasant	93	177	401	132	125	3.02	1.13
Harmful–Beneficial	105	93	366	223	141	3.22	1.16
Worthless–Useful	112	98	336	240	142	3.22	1.19
**Subjective Norms (α = 0.72)**							
Most people who are important to me think that online physical education during the COVID-19 period is effective	90	246	225	271	96	3.04	1.17
It is expected of me that I teach physical education online during the COVID-19 period	34	93	190	370	241	3.74	1.06
The people in my life whose opinions I value would agree that online physical education during the COVID-19 period is effective	87	206	248	317	70	3.08	1.11
I feel under social pressure to teach physical education online during the COVID-19 period	130	221	271	246	60	2.88	1.14
**Perceived Behavioral Control (α = 0.66)**							
If I wanted to, I could teach physical education online during the COVID-19 period	64	103	154	402	205	3.63	1.15
For me, to teach physical education online during the COVID-19 period is easy	134	283	206	251	53	2.79	1.16
I believe I have complete control over teaching physical education online during the COVID-19 period	91	203	215	313	106	3.15	1.18
It is mostly up to me whether or not I teach physical education online during the COVID-19 period	223	288	216	167	34	2.46	1.14
**Section 2 : Constraints**	**1**	**2**	**3**	**4**	**5**		
**Interpersonal (α = 0.74)**							
My colleagues do not support delivering physical education courses remotely during the COVID-19 period	153	318	253	149	53	2.60	1.12
Students do not accept participating in physical education courses remotely during the COVID-19 period	94	336	196	247	54	2.81	1.11
Students’ parents do not facilitate their participation in physical education activities during the COVID-19 period	84	261	239	276	67	2.97	1.11
**Intrapersonal (α = 0.65)**							
I don’t know how to teach physical education remotely during the COVID-19 period	315	352	171	70	19	2.05	1.00
I believe it is not possible to teach physical education during the COVID-19 period	296	342	149	100	41	2.19	1.13
I don’t like teaching physical education remotely during the COVID-19 period	112	237	193	240	146	3.08	1.27
**Structural (α = 0.76)**							
There aren’t enough opportunities to deliver physical education lessons remotely during the COVID-19 period	164	286	211	196	71	2.70	1.20
Our institution (university/school) has provided enough support to deliver physical education courses during the COVID-19 period (reverse-coded)	177	228	212	202	109	2.83	1.29
The government did not prepare the environment for participation in physical education remotely during the COVID-19 period	141	138	302	225	122	3.05	1.23
Students do not have access to the required equipment to participate in physical education activities remotely during the COVID-19 period	87	206	188	293	154	3.24	1.23
Students do not have access to a proper place to participate in physical education activities during the COVID-19 period	67	211	196	329	125	3.25	1.16
I don’t have the technical equipment (computer, camera, microphone, etc.) required to teach physical education remotely during the COVID-19 period	338	310	142	106	32	2.12	1.13

**Table 3:** Employment and teaching experiences before and during the COVID-19 pandemic.

Variable	Frequency	Percentage
**Employment status before the COVID-19 pandemic**		
Employed full-time	848	91.4
Employed part-time	52	5.6
Unemployed	21	2.3
Retired	2	0.2
Unable to work	5	0.5
**Change in employment status during the pandemic**		
No change	693	74.7
Changed	191	20.6
I don’t know	21	2.3
Prefer not to answer	16	1.7
**Experience in using online teaching service prior to the COVID-19 pandemic**		
Yes	281	30.3
No	647	69.7
**Taught physical education during the COVID-19 pandemic**		
Yes	761	82.0
No	167	18.0
**Preferred teaching method during the COVID-19 period**		
Online education	252	27.2
Face-to-face education	402	43.3
Hybrid education	274	29.5

**Table 4:** One-way ANOVA results and descriptive statistics by country.

Variable	Country	*N*	Mean	SD	S.E.	*F*	*p*-Value
Overall Intention	Turkey *	215	3.21	1.30	0.09	19.08	<0.001
	Malaysia *	141	3.07	0.97	0.08		
	US ^1^	352	3.74	0.89	0.05		
	China *	153	3.23	0.78	0.06		
	Philippines ^1^	67	3.79	0.97	0.12		
	Total	928	3.44	1.04	0.03		
Overall Attitude	Turkey	215	2.66	1.25	0.08	17.43	<0.001
	Malaysia *	141	3.10	0.583	0.05		
	US *^,1^	352	3.26	0.66	0.04		
	China *^,2^	153	3.32	0.39	0.03		
	Philippines ^1,2^	67	3.51	0.69	0.08		
	Total	928	3.12	0.84	0.03		
Overall SN	Turkey *	215	3.02	0.77	0.05	6.73	<0.001
	Malaysia *^,2^	141	3.14	0.75	0.06		
	US ^1^	352	3.36	0.65	0.03		
	China *	153	2.97	0.36	0.03		
	Philippines ^1,2^	67	3.37	0.70	0.09		
	Total	928	3.19	0.68	0.02		
Overall PBC	Turkey	215	3.31	1.07	0.07	7.02	<0.001
	Malaysia *	141	3.01	0.78	0.07		
	US *	352	2.85	0.71	0.04		
	China *	153	2.10	0.52	0.04		
	Philippines *	67	2.87	0.77	0.09		
	Total	928	3.01	0.86	0.03		
Intrapersonal Constraints	Turkey *	215	2.40	1.08	0.07	16.07	<0.001
	Malaysia	141	3.05	0.76	0.06		
	US *	352	2.30	0.75	0.04		
	China *	153	2.38	0.69	0.06		
	Philippines *	67	2.20	0.84	0.10		
	Total	928	2.44	0.88	0.03		
Interpersonal Constraints	Turkey *	215	2.81	1.17	0.08	9.05	<0.001
	Malaysia	141	3.18	0.82	0.07		
	US *	352	2.75	0.78	0.04		
	China *	153	2.74	0.76	0.06		
	Philippines	67	2.33	0.71	0.09		
	Total	928	2.80	0.91	0.03		
Structural Constraints	Turkey *	215	2.55	1.17	0.08	14.05	<0.001
	Malaysia ^1^	141	3.25	0.61	0.05		
	US ^2^	352	2.94	0.60	0.03		
	China *	153	2.66	0.66	0.05		
	Philippines ^1,2^	67	3.13	0.58	0.07		
	Total	928	2.87	0.81	0.03		

Notes: *, ^1^, and ^2^ show the differences between the groups based on the Tukey’s HSD post hoc test results.

**Table 5:** Regression associations.

Predictor	Dependent Variable	*Β*	*p*-Value	S.E.	Indirect Effect
Attitude	Intention	0.37	<0.001	0.078	--
Subjective Norms	Intention	0.08	<0.05	0.106	--
PBC	Intention	0.49	<0.01	0.130	--
Constraints	Attitude	−0.81	<0.001	0.070	--
Constraints	Subjective Norms	−0.49	<0.001	0.034	--
Constraints	PBC	−0.716	<0.001	0.049	--
Constraints	Intention	--	--		0.69

Note: R^2^ _Intention_ = 0.67; R^2^ _Attitude_ = 0.66; R^2^ _SN_ = 0.24; R^2^ _PBC_ = 0.51.

**Table 6:** Model fit indices.

	χ^2^ (*df*)	IFI	NFI	GFI	CFI	RMR	RMSEA
Model	263.76 (38)	0.956 **	0.949 **	0.957 **	0.956 **	0.043 ***	0.080 ****

Note: Criteria for the fit model indices: ** IFI, NFI, GFI, and CFI > 0.90; *** RMR < 0.05; **** RMSEA close to 0.05.

## Data Availability

The raw data supporting the conclusions of this article will be made available by the authors on request.
